# Biliary protozoa in a dog with acute cholangiohepatitis fed a raw food diet

**DOI:** 10.1111/jvim.16565

**Published:** 2022-09-30

**Authors:** Frederik Allan, Damer Blake, Zoe Miller, David Church

**Affiliations:** ^1^ Department of Veterinary Clinical Sciences, Royal Veterinary College University of London Hertfordshire UK; ^2^ Pathobiology and Population Sciences Royal Veterinary College North Mymms UK

**Keywords:** biliary protozoa, cholangiohepatitis, *Hammondia*, raw‐feeding

## Abstract

A 1‐year 11‐month intact female Alaskan Malamute fed a raw food diet was referred to the Queen Mother Hospital for Animals for further investigation of hyporexia and increased hepatobiliary enzyme activities. Clinicopathological and imaging findings were consistent with cholangiohepatitis, with coccidial zoites identified on bile cytology. Polymerase chain reaction and amplicon sequencing from the bile identified *Hammondia heydorni*, a Sarcocytid coccidial protozoa with an obligate 2‐host life cycle. The dog was treated with clindamycin, marbofloxacin, ursodeoxycholic acid (UDCA) and S‐adenosylmethionine/silybin with complete clinical and biochemical resolution documented after 6 weeks. Infection with *Hammondia* spp. should be considered in patients receiving raw food diets in which coccidial zoites are identified in the bile, but the pathogenic potential of this organism is unknown and the possibility of its presence as a commensal cannot be discounted.

## 1 INTRODUCTION

*Hammondia heydorni* is a cyst‐forming coccidian parasite with a 2‐host life cycle. Definitive host infection occurs by ingestion of an intermediate host.^1^, ^2^ Little is known about the pathogenicity of the Sarcocystid coccidian protozoa *H. heydorni* in dogs, but diarrhea is the most frequently reported clinical sign in clinical cases.^3^, ^4^ Other Sarcocystid coccidian protozoa have been associated with hepatitis in dogs,^5^, ^6^ with the presence of *Hammondia* species identified in bile from a dog with acute hepatitis^7^ and *Isospora* spp. also documented in canine bile,^8^ but the clinical relevance of these biliary protozoa is unclear.

We report the first case in which coccidial zoites of *H. heydorni* have been documented in the bile of a dog that was fed raw food with clinical, clinicopathological and imaging findings consistent with cholangiohepatitis.

## 2 CASE DESCRIPTION

A 1‐year 11‐month, intact female Alaskan Malamute presented for further investigation of hyporexia, pigmenturia and increased hepatobiliary enzyme activities identified before referral. The dog had no history of travel outside of the United Kingdom and was fed a commercial raw food diet. On presentation, physical examination disclosed icteric sclera, mild cranial abdominal discomfort, and an increased body condition score of 8/9, but was otherwise unremarkable. Initial investigations included a CBC, serum biochemistry profile and abdominal ultrasonography. The CBC identified a mild neutrophilia (14.22 × 10^9^/L; reference interval, 3.5‐11.5 × 10^9^/L) with reactive lymphocytes. Serum biochemistry documented markedly increased alkaline phosphatase (ALP) activity (6232 U/L; reference interval, 0‐130 U/L), alanine aminotransferase (ALT) activity (2481.6 U/L; reference interval, 19.8‐124 U/L), total bilirubin concentration (59.9 μmol/L; reference interval, 0.1‐4.2 μmol/L) and C‐reactive protein (CRP) concentration (121.3 mg/L; reference interval, 0‐10 mg/L) along with mild increases in cholesterol concentration (11.95 μmol/L; reference interval, 3.2‐6.2 μmol/L) and creatine kinase (CK) activity (677 U/L; reference interval, 67‐446 U/L), and a mild decrease in urea concentration (2.4 mmol/L; reference interval, 3.1‐10.1 mmol/L). Leptospira microscopic agglutination testing (MAT) was not consistent with active leptospirosis and urine PCR was negative for leptospires.

Abdominal ultrasonography identified mildly heterogenous liver parenchyma with mild rounding of the right liver margins, moderate dilatation of the common bile duct with hyperechoic mildly thickened walls, mild hepatic lymphadenopathy and a scant amount of peritoneal fluid adjacent to the gallbladder. Fine needle aspirates (FNA) of the liver and cholecystocentesis were performed without complication.

**FIGURE 1:**
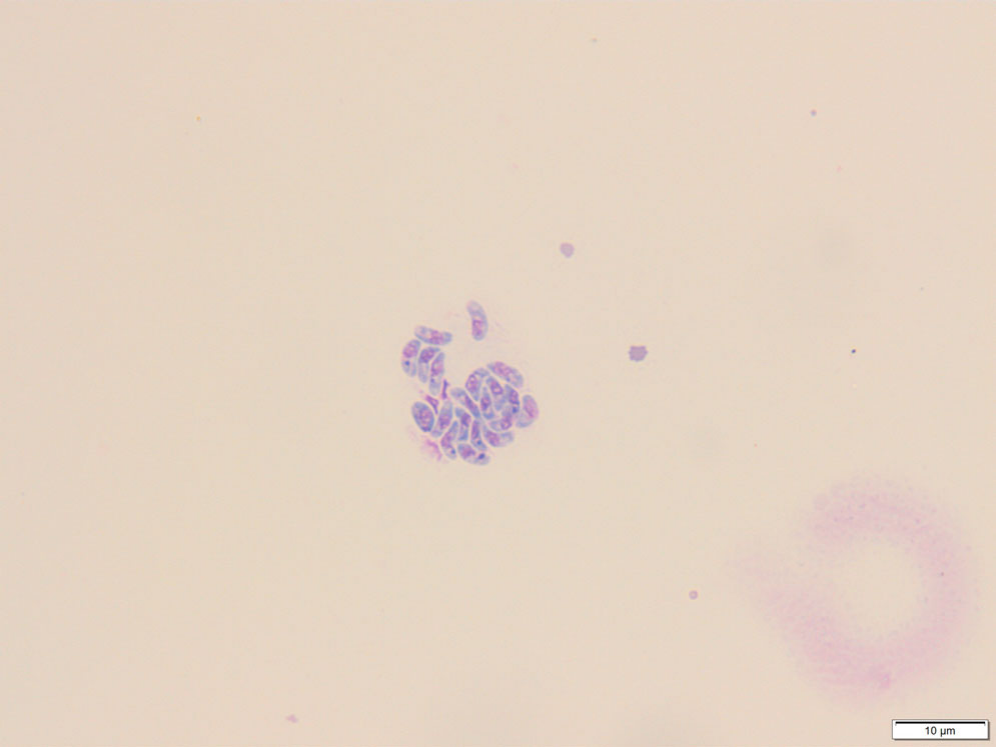
Image of a cluster of grouped crescent‐shaped *Hammondia heydorni* tachyzoites identified on cytocentrifuged preparation bile cytology at ×100 magnification

Liver FNA identified moderate numbers of segmented neutrophils seen focally throughout hepatocyte clusters, high numbers of hepatocytes containing lipid vacuoles, and moderate numbers of bile casts, findings consistent with neutrophilic inflammation, vacuolar degeneration (lipid type) and cholestasis. Bile cytology on both direct and cytocentrifuged preparations documented low to moderate numbers of crescent‐shaped zoites with light to moderate blue cytoplasm and central to pericentral nuclei, both individually and in clumps (Figure 1). Bile culture failed to identify any bacterial isolates. After cytological identification of coccidial zoites, serology for *Toxoplasma* and *Neospora* was performed. *Toxoplasma* Immunoglobulin M (160; reference interval, <20) and Immunoglobulin G (>800; reference interval, <50) titers were strongly indicative of exposure, as was *Neospora* serology (Immunoglobulin G immunofluorescence >1600; reference interval, <100). The PCR of the patient's bile however was negative for *Toxoplasma* and *Neospora* (Langford Veterinary Services, Bristol, UK). The dog was treated with a 4‐week course of marbofloxacin (3.89 mg/kg q24h PO [Marbocyl, Vetoquinol, UK]), a 4‐week course of clindamycin (31.2 mg/kg q12h PO [Antirobe, Zoetis, USA]), a 4‐week course of ursodeoxycholic acid (UDCA; 11.7 mg/kg q24h PO [Destolit, Norgine, UK]), a 4‐week course of S‐Adenosylmethionine/Silybin (16.6 mg/kg q24h PO [Denamarin, Protexin, UK]) and it was recommended to transition the dog onto a commercial cooked hydrolyzed protein diet and to reassess the dog in 4 weeks. One week after presentation, repeat CBC was normal and serum biochemistry indicated marked improvement in ALP (3045 U/L; reference interval, 0‐130 U/L) and ALT (818.3 U/L; reference interval, 19.8‐124 U/L) activities, and total bilirubin (7.5 μmol/L; reference interval 0.1‐4.2 μmol/L) and CRP (18.4 mg/L; reference interval 0‐10) concentrations, but these results had not normalized. The patient's hyporexia and pigmenturia had resolved. At 6 weeks, repeat CBC and serum biochemistry documented complete resolution of the patient's previously increased ALP (109 U/L; reference interval, 0‐130 U/L) and ALT (52 U/L; reference interval, 19.8‐124 U/L) activities and total bilirubin (2.9 μmol/L; reference interval, 0.1‐4.2 μmol/L) and CRP (0.6 mg/L; reference interval, 0‐10 mg/L) concentrations and the owner reported no clinical concerns.

## 3 MOLECULAR IDENTIFICATION OF *H. HEYDORNI*

Total genomic DNA was extracted from bile using a Qiagen DNeasy Blood & Tissue kit as recommended by the manufacturer (Qiagen, Hilden, Germany). Polymerase chain reaction followed by amplicon sequencing targeting coccidial 28S ribosomal DNA (rDNA) was performed using the primers KL‐P1Fa (5′‐TACCCGCTGAACTTAAGCAT‐3′) and KL‐P2Rb (5′‐TGCTACTACCACCAAGATCTGC‐3′) as described previously.^9^ Sequences were manually curated using CLC Main Workbench (version 8.0.1) and annotated following comparison using BLASTn (https://blast.ncbi.nlm.nih.gov/Blast.cgi), identifying *H. heydorni* by comparison with GenBank reference sequence AF159240 (100% query coverage, 99.79% identity).^10^ The consensus sequence is available under GenBank accession number ON738675.

## 4 DISCUSSION

This case is the first report of *H. heydorni* identified in the bile of a dog presenting with clinical signs, clinicopathological features and imaging findings consistent with acute cholangiohepatitis. A single report exists describing biliary *Hammondia* spp. in a dog with acute hepatitis,^7^ but the previous study could not differentiate between *H. heydorni* or *Hammondia triffittae*.

Sarcocystidae species have been described as a causative factor in hepatitis affecting companion animals, namely *Toxoplasma* in cats, and *Sarcocystis* and *Neospora* in dogs. *Hammondia* was historically not believed to be associated with disease in dogs, but recent literature suggests this organism may be an emerging pathogen.^1^, ^3^, ^4^ Hammondiasis has been reported infrequently, often is associated with diarrhea,^4^, ^11^ and has been identified in dogs fed raw meat.^11^ Infection secondary to feeding a raw meat diet in the dog described here would be compatible with the obligate 2‐host life cycle of *Hammondia*,^2^ with small rodents and ruminants usually acting as intermediate hosts, and dogs and other canids as definitive hosts in natural infection. Further evidence supporting the fact that consumption of a raw meat diet could have led to infection in the dog described here is that *H. heydorni*‐like oocysts have been documented in the feces of dogs fed musculature of infected cattle, sheep, goats, roe deer, and other herbivores.^2^

Feeding of raw diets is a rapidly growing trend with substantial concern regarding both the development of food‐borne disease in pets and the risk to humans handling raw food products and in contact with animals fed such diets and their environment.^12^ Several dangerous pathogens with zoonotic capacity have been identified in commercial raw food diets and raw meat, including extended‐spectrum β‐lactamase producing *Enterobacterales*, *Salmonella* species, *Clostridium perfringens* and several other bacterial and protozoal pathogens.^12^, ^13^, ^14^, ^15^ Biliary *H. heydorni* in the dog described here may represent a novel unusual infectious disease related to feeding of raw meat, but both the source of infection and the pathogenicity of *H. heydorni* in our case cannot be definitively proven.

A previous report in which *Hammondia* species were identified in the bile of a dog with acute hepatitis is similar to the case presented here,^7^ in which marked increases in ALP and ALT activities were evident along with cytologic evidence of hepatic neutrophilic inflammation and biliary coccidial zoites. However, a limitation of the former report was the inability to distinguish *H. heydorni* from *H. triffittae*. Both the aforementioned case and our case had biochemical and clinical resolution after treatment, having both received clindamycin, S‐Adenosylmethionine/Silybin and a fluoroquinolone. Ursodeoxycholic acid was utilized in our dog for its cholerectic action,^16^ given the ultrasonographic and biochemical evidence of cholestasis.

Interestingly, serology for *Toxoplasma* and *Neospora* were positive in both our dog and in another report^7^ despite negative PCR, and subsequent identification of *Hammondia*. This finding suggests a likely degree of cross‐reactivity in canine assays between Sarcocystidiae species, similar to what has been observed in ruminants and rodents.^17^, ^18^, ^19^

It is not possible to conclude that hammondiasis was a definitive contributing factor to the cholangiohepatitis in our case, and clinical improvement could be attributed to treatment with marbofloxacin, ursodeoxycholic acid or S‐Adenosylmethionine/Silybin or to spontaneous resolution, rather than resolution of hammondiasis secondary to treatment with clindamycin. A limitation of our report is that repeat cholecystocentesis to document persistence or resolution of *Hammondia* was not performed. It is possible *Hammondia* may have been incidentally detected as a commensal organism that refluxed into the bile from the intestine, was present as an opportunistic pathogen secondary to concurrent disease, or could indeed have acted as a primary causative pathogen. Despite this uncertainty, identification of biliary protozoa is a rarely reported and interesting finding, and ours is the first report of definitively identified *H. heydorni* in canine bile.

Clinical and clinicopathological resolution was achieved in this dog with normalization of bilirubin concentration and hepatobiliary enzyme activities 44 days postdiagnosis. Repeat cholecystocentesis was not performed to confirm absence or persistence of *H. heydorni* because of lack of benefit in a clinically healthy patient.

To conclude, we describe the first report of confirmed *H. heydorni* in bile of a dog fed a raw food diet with suspected cholangiohepatitis. This case adds to the body of evidence surrounding *H. heydorni* as an emerging organism of interest, although the clinical relevance of identification of this Sarcocystid organism in this case is unknown.

## CONFLICT OF INTEREST DECLARATION

Authors declare no conflict of interest.

## OFF‐LABEL ANTIMICROBIAL DECLARATION

Authors declare no off‐label use of antimicrobials.

## INSTITUTIONAL ANIMAL CARE AND USE COMMITTEE (IACUC) OR OTHER APPROVAL DECLARATION

Authors declare no IACUC or other approval was needed.

## HUMAN ETHICS APPROVAL DECLARATION

Authors declare human ethics approval was not needed for this study.

## References

[1] Jie HJ , Yu M , Fen YY , et al. First isolation of *Hammondia heydorni* from dogs in China. Vet Parasitol. 2013;197:43‐49. doi:10.1016/J.VETPAR.2013.04.035 23731857

[2] Dubey JP , Barr BC , Barta JR , et al. Redescription of *Neospora caninum* and its differentiation from related coccidia. Int J Parasitol. 2002;32:929‐946. doi:10.1016/S0020-7519(02)00094-2 12076623

[3] Reichel MP , Ellis JT , Dubey JP . Neosporosis and hammondiosis in dogs. J Small Anim Pract. 2007;48:308‐312. doi:10.1111/J.1748-5827.2006.00236.X 17547641

[4] Steffl M , Nautscher N . Detection of *Hammondia heydorni*‐like oocysts in feces of a dog with recurrent diarrhea. Tierarztliche Praxis Ausgabe K: Kleintiere—Heimtiere. 2019;47:189‐192. doi:10.1055/A-0890-2350 31212351

[5] Allison R , Williams P , Lansdowne J , Lappin M , Jensen T , Lindsay D . Fatal hepatic sarcocystosis in a puppy with eosinophilia and eosinophilic peritoneal effusion. Vet Clin Pathol. 2006;35:353‐357. doi:10.1111/J.1939-165X.2006.TB00148.X 16967426

[6] Fry DR , McSporran KD , Ellis JT , Harvey C . Protozoal hepatitis associated with immunosuppressive therapy in a dog. J Vet Intern Med. 2009;23:366‐368. doi:10.1111/J.1939-1676.2008.0263.X 19192151

[7] Irvine KL , Walker JM , Friedrichs KR . Sarcocystid organisms found in bile from a dog with acute hepatitis: a case report and review of intestinal and hepatobiliary *Sarcocystidae* infections in dogs and cats. Vet Clin Pathol. 2016;45:57‐65. doi:10.1111/VCP.12330 26870918

[8] Peters LM , Glanemann B , Garden OA , Szladovits B . Cytological findings of 140 bile samples from dogs and cats and associated clinical pathological data. J Vet Intern Med. 2016;30:123‐131. doi:10.1111/JVIM.13645 26566964 PMC4913648

[9] Shadbolt T , Pocknell A , Sainsbury AW , Egerton‐Read S , Blake DP . Molecular identification of *Sarcocystis wobeseri*‐like parasites in a new intermediate host species, the white‐tailed sea eagle (*Haliaeetus albicilla*). Parasitol Res. 2021;120:1845‐1850. doi:10.1007/S00436-021-07103-0 33666756

[10] Mugridge NB , Morrison DA , Heckeroth AR , Johnson AM , Tenter AM . Phylogenetic analysis based on full‐length large subunit ribosomal RNA gene sequence comparison reveals that *Neospora caninum* is more closely related to *Hammondia heydorni* than to *Toxoplasma gondii* . Int J Parasitol. 1999;29:1545‐1556. doi:10.1016/S0020-7519(99)00150-2 10608441

[11] Dubey JP , Ross AD , Fritz D . Clinical *Toxoplasma gondii*, *Hammondia heydorni*, and *Sarcocystis* spp. infections in dogs. Parassitologia. 2003;45:141‐146. 15267102

[12] Lejeune JT , Hancock DD . Public health concerns associated with feeding raw meat diets to dogs. J Am Vet Med Assoc. 2001;219:1222‐1225. doi:10.2460/JAVMA.2001.219.1222 11697364

[13] Strohmeyer RA , Morley PS , Hyatt DR , Dargatz DA , Scorza AV , Lappin MR . Evaluation of bacterial and protozoal contamination of commercially available raw meat diets for dogs. J Am Vet Med Assoc. 2006;228:537‐542. doi:10.2460/JAVMA.228.4.537 16478425

[14] Cole SD , Healy I , Dietrich JM , Redding LE . Evaluation of canine raw food products for the presence of extended‐spectrum beta‐lactamase‐ and carbapenemase‐producing bacteria of the order *Enterobacterales* . Am J Vet Res. 2022;83(9). doi:10.2460/AJVR.21.12.0205 PMC9661483 35895774

[15] Weese JS , Rousseau J , Arroyo L . Bacteriological evaluation of commercial canine and feline raw diets. Can Vet J. 2005;46:513‐516. 16048011 PMC1140397

[16] Allan F , Watson PJ , McCallum KE . Clinical features and outcomes in 38 dogs with cholelithiasis receiving conservative or surgical management. J Vet Intern Med. 2021;35:2730‐2742. doi:10.1111/JVIM.16284 34714561 PMC8692201

[17] Christie E , Dubey JP . Cross‐immunity between *Hammondia* and *Toxoplasma* infections in mice and hamsters. Infect Immun. 1977;18:412‐415. doi:10.1128/IAI.18.2.412-415.1977 411757 PMC421248

[18] Riahi H , Leboutet MJ , Bouteille B , Dubremetz JF , Dardé ML . *Hammondia hammondi* organelle proteins are recognized by monoclonal antibodies directed against organelles of *Toxoplasma gondii* . J Parasitol. 1999;85:580‐583. doi:10.2307/3285803 10386461

[19] Munday BL , Dubey JP . Serological cross‐reactivity between *Hammondia hammondi* and *Toxoplasma gondii* in experimentally inoculated sheep. Aust Vet J. 1986;63:344‐345. doi:10.1111/J.1751-0813.1986.TB02886.X 3541885

